# Molecular cloning and characterization of the endothelin 3 gene in black bone sheep

**DOI:** 10.1186/s40104-018-0272-y

**Published:** 2018-06-25

**Authors:** Hesham Y. A. Darwish, Yuanyuan Zhang, Kai Cui, Zu Yang, Deping Han, Xianggui Dong, Huaming Mao, Weidong Deng, Xuemei Deng

**Affiliations:** 1 National Engineering Laboratory for Animal Breeding and Key Laboratory of Animal Genetics, Breeding, and Reproduction of the Ministry of Agriculture, China Agricultural University, Beijing, 100193 China; 2 Animal Production Research Institute, Agricultural Research Center, Ministry of Agriculture and Land Reclamation, Giza, 12618 Egypt; 3 College of Veterinary Medicine, China Agricultural University, Beijing, 100193 China; 4 College of Animal Science and Technology, Yunnan Agricultural University, Kunming, 650201 China

**Keywords:** Black pigmentation, Expression level, Sequence analysis, SNP

## Abstract

**Background:**

Black bone sheep was first discovered in Yunnan province of China in 1970, with unique black pigmentation on the body and internal organs. Endothelin 3 (*EDN3*) has been known as a key gene causing hyperpigmentation in black bone chicken, the Silky fowl.

**Methods:**

In this study, *EDN3* was employed as a candidate gene for regulating black color pigmentation. First, *EDN3* was cloned from sheep to obtain the full-length cDNA by using the rapid amplification of cDNA ends (RACE). Genomic *EDN3* was screened and a total of thirty predicted single nucleotide polymorphisms (SNPs) were genotyped for allele and genotype frequency analysis in a case-control study involving two black bone sheep populations. Genomic copy number analysis of *EDN3* in sheep was conducted to measure the variation in copy number. *EDN3* expression levels were observed among the groups in adult liver, lymph node, and kidney tissues, as well as embryo kidney samples. Also, among the tissues of black bone and non-black bone sheep.

**Results:**

The size of the full-length cDNA was 1,578 bp, which included 426 bp of 5′-untranslated region (5′-UTR), an open reading frame (ORF) of 639 bp encoding a protein of 212 amino acids, and a 3′-UTR of 513 bp. Genotype and allele frequencies of all the discovered SNPs were found insignificantly different in black bone and non-black bone sheep (*P* > 0.05). Genomic copy number analysis of *EDN3* in sheep revealed no significant difference between the two sheep groups. No significant variations were found in the adult liver and kidney embryo samples. However, the expression in lymph node and kidney tissue was significantly higher in black bone sheep than that in non-black bone sheep (*P* < 0.05). Significant variations in the *EDN3* expression levels were observed among the tissues of non-black bone sheep.

**Conclusions:**

The findings of the present study indicate that unlike in Silky chickens, *EDN3* is not responsible for hyperpigmentation but may play a key functional role in immune and excretory systems of black bone sheep.

**Electronic supplementary material:**

The online version of this article (10.1186/s40104-018-0272-y) contains supplementary material, which is available to authorized users.

## Background

Black bone sheep is a native Chinese sheep breed, discovered in Yunnan province of China in 1970 and characterized by black pigmentation across the body and on internal organs. The value of the sheep was not recognized until 2001 when black bone sheep were identified by Chinese scientists [1]. The pigmentation pattern caused by increasing levels of a black substance was found similar to that in Silky fowl, a famous domestic fowl that originated in China thousand years ago [2, 3]. Analysis of infrared spectrum of the pigment isolated from tissues of both the black bone sheep and Silky fowl was conducted to investigate this similarity. Although there was a slight difference, an overall similarity in the spectral pattern in the two species was observed, with eumelanin being the main pigment [2]. To date, more than 10 genes involved in melanogenesis have been identified and cloned, for example, tyrosinase (*TYR*), tyrosinase-related protein 1 (*TRP1*), tyrosinase-related protein 2 (*TRP2*) [4], microphthalmia-associated transcription factor (*MITF*) [5], and melanocortin 1 receptor (*MC1R*) [6]. Moreover, some genes are involved in migration and survival of melanocytes during development, for instance, KIT proto-oncogene receptor tyrosine kinase (*Kit*), Kit ligand 1 (*Kit l*) [7], *EDN3*, and endothelin receptor type B (*EDNRB*) [8], any loss in the functions of these genes results in the loss of skin pigmentation [9]. The studies used the candidate genes *TYR* and *MC1R* to investigate the black traits of black bone sheep reported that no causative association of these genes with the trait [1, 10]. Endothelins are peptides that constrict blood vessels and raise blood pressure [11]. The endothelin family comprises three ligands (EDN1, EDN2, and EDN3) and two types of currently known receptors endothelin receptor A (EDNRA) and EDNRB. *EDN3* produces signals through EDNRB and affects melanocyte proliferation and differentiation as well as enteric ganglia development [12]. In chickens, studies have illustrated that two duplicated genomic regions are found to be associated with dermal hyperpigmentation phenotype or fibromelanosis (FM). The first region contained *EDN3* gene and several other known coding elements, whereas the second region did not contain any regulatory or known coding elements. Each region was longer than 100 kb, joined to the other in an inverted manner and separated by 417 kb on wild type chicken chromosome 20 [3]. The findings of Shinomiya et al. [13] revealed that *EDN3* was duplicated in Silky chickens, which was not the case in other breeds having non-black bone. The higher gene expression resulting from gene duplication might have caused the hyperpigmentation in the connective tissues and internal organs of Silky chickens. Han et al. [14] agreed with Dorshorst and Shinomya et al. [3, 13] when observed that Silky chicken and Xichuan black bone chicken show copy number variations (CNVs) at the *EDN3* locus in which the dermal hyperpigmentation of these two Chinese local chicken breeds also resulted from a CNV in this region. More recent study was in accordance with the previous findings and reported that, beside Chinese silky chicken, other breeds such as Ayam Cemani in Indonesia, Black H Mong in Vietnam, and Svarthona in Sweden have also exhibited the duplicated region in *EDN3* gene [15]. Mutations in *EDN3* gene in human lead to Hirschsprung disease, a disorder that causes severe constipation or blockage of the intestine due to the lack of enteric nerves [16].

The main objective of the present study was to investigate the molecular mechanism of the dark pigmentation in black bone sheep. We built our hypothesis on considering *EDN3* as the candidate gene for the trait of interest as its obvious key role involved in hyperpigmentation pathway in Silky fowl, which has similar black pigmentation distribution patterns in the body as in black bone sheep.

## Methods

### Experimental animals

**Fig. 1:**
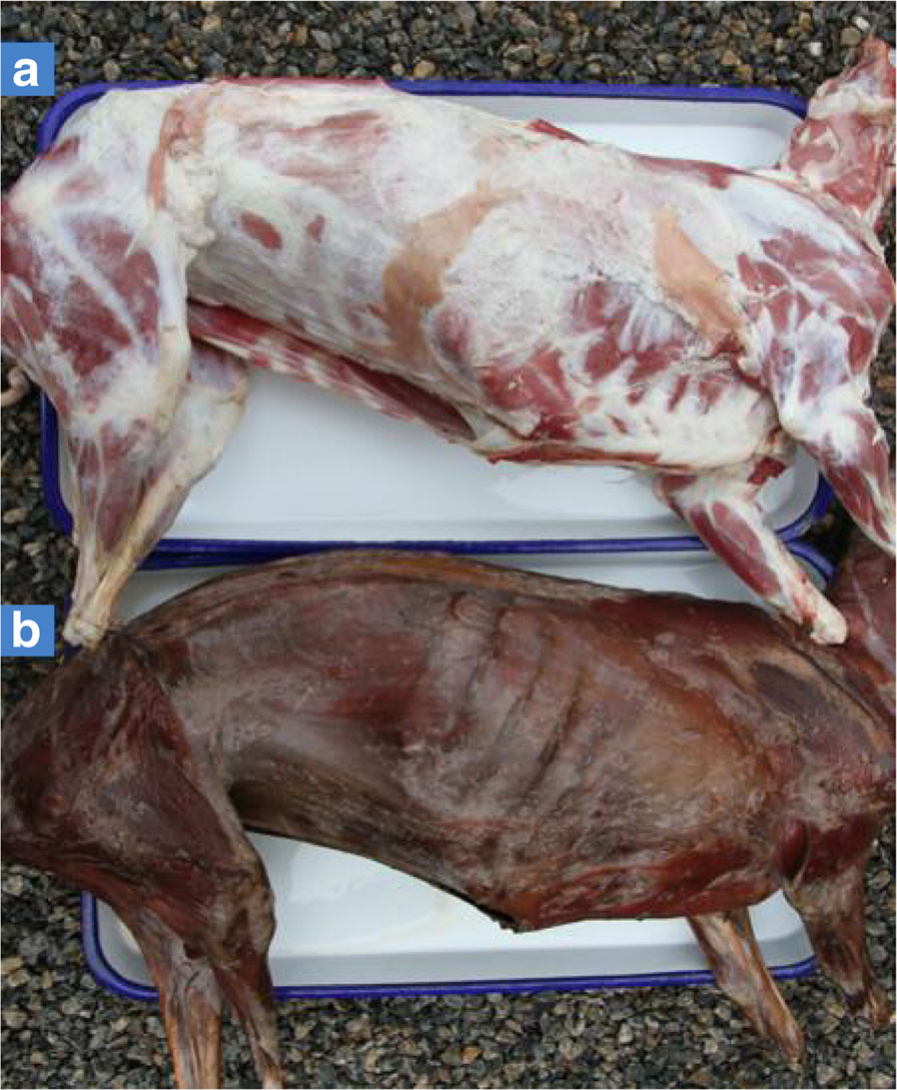
Different pigmentation patterns displayed after slaughtering of black bone and non-black bone sheep. **a** Non-black bone sheep. **b** Black bone sheep

Ear samples were collected from the black bone sheep (*n* = 110) and non-black bone sheep (*n* = 94) populations for detection of single nucleotide polymorphisms (SNPs) in *EDN3*. Two different populations were involved in the association study which were designed crossbreeding population and random local population. The designed crossbreeding population consisted of black bone (*n* = 22) and non-black bone (*n* = 63) sheep that came from a mating of indigenous Lanping black bone male sheep with the introduced Suffolk female sheep. The random local population consisted of Lanping black bone sheep individuals (*n* = 88) and Lanping non-black bone sheep (*n* = 31) obtained by natural mating of indigenous Lanping black bone and Lanping non-black bone sheep. All the animals had free access to food and water, and were raised under the same conditions. Six adult individuals, each of black bone and non-black bone types from the random local population, were sacrificed when they were 24-month-old (Fig. 1). Liver, kidney, and lymph node samples from the adult sheep were collected, snap-frozen in liquid nitrogen, and stored at − 80 °C until use. Three more black bone and three more non-black bone pregnant ewes from the same random local population were sacrificed to obtain the embryo samples.

### Reverse transcription PCR (RT-PCR) and cloning of *EDN3* gene in sheep

Total RNA was extracted from the liver sample using TRIZOL® Reagent (Invitrogen, San Diego, CA, USA), following the manufacturer’s protocol. The first strand of cDNA was synthesized from 1 to 2 μg of purified total RNA using the Promega ImProm-IITM Reverse Transcription System (Beijing, China). Primers F: 5′-CGCTCTGAAGTTTGTGACG-3′ and R: 5′- CAATGAATGCGTTTCCGAGATA-3′ were designed using Primer Premier 5.0 software (Premier Biosoft International, Palo Alto, CA, USA) based on the conserved sequence of bovine *EDN3* gene deposited in the GenBank database (Accession No: BC148051.1).

5′- and 3′-rapid amplification of cDNA ends (RACE) of the cDNA prepared from the liver tissue was performed to obtain the full-length sheep *EDN3* cDNA using Gene Racer Kit (full-length, RNA ligase-mediated rapid amplification of 5′ and 3′ cDNA ends, Invitrogen), according to the manufacturer’s protocol. PCR was performed using a total volume of 50 μL as follows: 25 μL 2 × Taq PCR mix, 1 μL GeneRacer primer (supplied with the kit), 1 μL gene specific primer (GSP), 1 μL cDNA, and 22 μL ddH_2_O. The PCR reaction program initially started with one cycle of denaturation at 94 °C for 2 min, followed by 5 cycles at 94 °C for 30 s, 72 °C for 1 min, 5 cycles at 94 °C for 30 s, 70 °C for 1 min, 25 cycles at 94 °C for 30 s, 65 °C for 30 s, 72 °C for 1 min, one cycle of final elongation at 72 °C for 10 min, and 4 °C to terminate the reaction. The RACE PCR products were then run onto 1% agarose gel electrophoresis. The obtained PCR products were diluted and used as a template to run touchdown PCR by using nested primers. The PCR products were cloned into PMD19-T vector and sequenced in two directions. Gene specific primers and nested primers shown in Table 1 were designed using primer premier 5.0 software based on the sequence that was previously obtained (Accession no. KC857456).

**Table 1:** Primers used for 5′ and 3′ RACE

Primer name	Sequences (5′→3′)	T_a_, °C	PCR
5´ Gene specific primer	ACCGTCTCCTTGGTGTCCCCCTCCGAT	65	Normal - kit protocol
5´ Gene specific nested primer	ATCCTGCGGCGGAGGTCACAGCGAG	75	Touchdown
3´ Gene specific primer	GCTGAGGTGTTAGCCTTGACCAAATGC	65	Normal - kit protocol
3´ Gene specific nested primer	TGGAAAGGACTGATGTGCCAGCGAGAT	75	Touchdown

### Bioinformatics and sequence analysis

Gene analysis for cDNA sequences was conducted using GenScan software (http://genes.mit.edu/GENSCAN.html). Multiple sequence alignment of the coding domain sequence (CDS) from different species was conducted by ClustalW (http://www.ebi.ac.uk/Tools/msa/clustalw2). Protein secondary structure prediction method used was SOPMA (http://npsa-pbil.ibcp.fr/) [17]. The transmembrane domain was predicted by TMHMM Serverv 2.0 (http://www.cbs.dtu.dk/services/TMHMM-2.0) from the putative amino acid sequences. The protein molecular weight (Mw) and theoretical isoelectric point (pI) values were calculated by the Compute pI/Mw Tool (http://web.expasy.org/compute_pi). Protein localization sites in the cell were predicted by PSORT II Prediction (http://psort.hgc.jp). Phylogenetic and molecular evolutionary analyses of *EDN3* were conducted using MEGA version 4 [18].

### SNP screening

Genomic DNA was extracted from the ear tissues using the phenol–chloroform method, after proteinase-K digestion [19]. Two DNA pools were made up of black bone and non-black bone sheep, with five DNA samples in each pool. The DNA in each sample was adjusted to a final concentration of 50 ng/μL. In addition, DNA from one individual of black bone type having the most noticeable black phenotype as well as from non-black bone sheep was used in the study. PCR primers were designed to amplify the *EDN3* regions of ovine genomic DNA. Variations in primers spanned the 5′-flanking region, all exons, and partial introns. Primer sequences, targeted regions, and the amplicon size are shown in Table 2. Chromas Pro and DNAMAN6.0 were used to analyze the sequencing results.

**Table 2:** Primer pairs used to scan *EDN3* gene for polymorphism

Primer No.	Primer sequences (5′→3′)	Binding regions	Product size, bp	T_a_, °C	Location (UCSC)
1	F-ATTAGGTGAACGCTGACA R-CCCTCCAACTGCAGATGC	5 Flanking region, partial exon 1	579	56	61,296,366–61,296,944
2	F-CGCTCTGAAGTTTGTGACG R-AGACCCTTACACCCACCATC	Partial exon1, partial intron1	782	56	61,295,675–61,296,460
3	F-ACGCTGTGGAGTAAGTGAGA R-TAGAGACCAGGAAAGCAAAT	Partial intron1, exon 2, partial intron2	1,335	52	61,294,558–61,296,896
4	F-AGCCACCTTGTTTTACCG R-GCCCAAGTTCCCTAAAGC	Intron 2	1,268	52	61,293,086–61,294,353
5	F-TGGTAGGGTGGCAAGATA R-TCCAGGTCAAATGTAGGC	Intron 2	914	50	61,285,341–61,286,254
6	F-GTAGGAAGGCATCTTATTGG R-GAGTAACGCAGGTGAACG	Partial intron2, exon 3, partial intron3	1,231	50	61,275,666–61,276,905
7	F-CTCAGCCTTCGGAAACTAT R-CTCCTCTGCGTTTTTATTGT	Partial intron3, exon4, partial intron 4	946	50	61,274,254–61,275,198
8	F-CCAGACAAAGCAAGTAGG R-GAATGCGTTTCCGAGATA	Partial intron4, exon5	1,407	51	61,271,825–61,273,231

Note: UCSC genome browser on Feb.2010, ISGC *Ovis_aries* _1.0/oviAri. http://genome.ucsc.edu/cgi-bin/hgBlat

### SNP genotyping and association model

We searched the available online database (http://www.livestockgenomics.csiro.au/sheep/oar3.1.php) to detect more SNPs close to *EDN3* gene position on ovine chromosome 13. Genotyping of polymorphisms from genome screening and the online database was carried out using DNA samples extracted from ear samples of 110 black bone sheep and 94 non-black bone sheep by matrix assisted laser desorption-ionization time-of-flight mass spectrometry (MALDI-TOF MS) on the Mass ARRAY iPLEX Platform (Sequenom, San Diego, CA). Fisher’s exact test was used in the genotypic frequency distribution analysis.

### Genomic copy number variation analysis of *EDN3* in sheep

We collected genomic DNA samples from eight black bone sheep and eight non-black bone sheep to detect the *EDN3* copy number. All of these black bone and non-black bone sheep were from Lanping county in Yunnan province, China. To detect the copy number of *EDN3*, we performed genomic quantitative real-time PCR assays. The DNA concentrations of samples from eight black bone and eight non-black bone sheep were adjusted to 40 ng/μL in each reaction. Since *EDN3* was found to be a single copy gene in common sheep that did not possess any segmental duplication [20], *EDN3* target primers were designed from part of intron 2 and the primers were F: 5′- TGGGTGCAGATCAAGCTCAG-3′ and R: 5′- CAGTTGCACGGAGGTAGAGG-3′. Diacylglycerol O-acyltransferase 1 (*DGAT1*) was chosen as a reference gene with a single copy and the primers were F: 5′- TCAACGACTGGATGACTGCC-3′ and R: 5′- TTTCCCACTTGGGCCAGTTT-3′. Primers were designed using Primer Premier 5.0 software and validated by using standard curve, melting curve analysis, and a control sample without template. Series of dilution points from one genomic DNA were assigned for standard curve analysis. The quantitative real-time PCR (qRT-PCR) was conducted on a CFX96 Real-Time System (Bio-Rad, USA) with a total volume of 20 μL. Data were analyzed using ΔΔCt method to normalize the target Ct value with respect to the Ct value within the sample, and subsequently all the samples were normalized to a known calibrator sample which was one non-black bone sheep sample.

### Quantitative expression of *EDN3* mRNA in adult tissues

Total RNA was extracted from liver, lymph node, and kidney as described above. The quantitative expression of *EDN3* gene was performed using real-time PCR. Primers were designed using Primer Premier 5.0 software according to the previously acquired sequence. Gene specific primers were F: 5′-CTACAGAGGCAGCGGAAG-3′ and R: 5′-AAGCAGGCATCATCATCG-3′. The qRT-PCR was conducted with the following program: 95 °C for 30 s, 39 cycles at 95 °C for 10 s, 57 °C for 30 s, and a final extension step at 95 °C for 10 s on a CFX96 Real-Time System (Bio-Rad, USA). The house-keeping gene glyceraldehyde-3-phosphatedehydrogenase (*GAPDH*) was selected as internal control [21]. *GAPDH* primers were F: 5′-GTCCGTTGTGGATCTGACCT-3′ and R: 5′-TGCTGTAGCCGAATTCATTG-3′. The real-time PCR efficiency of each pair of primers was calculated using 5 points in a 5-fold dilution series of cDNA, which was used to construct a standard curve. Quantitative expression of gene was calculated using the 2¯^ΔΔCt^ method (ΔΔCt = ΔCt target gene −ΔCt housekeeping gene) [22].

### RT-PCR and qRT-PCR from embryo samples of black bone and non-black bone sheep

Total RNA was extracted from embryo kidney samples of black bone and non-black bone sheep. The primer pair used for RT-PCR was the same as used in real-time PCR. To compare the relative expression levels of *EDN3* between the black bone and non-black bone sheep embryos, we analyzed the intensity of the RT-PCR bands by ImageJ software (Rasband, W.S., ImageJ, U. S. National Institutes of Health, Bethesda, Maryland, USA, http://imagej.nih.gov/ij, 1997–2014). Furthermore, we calculated the expression quantity of *EDN3* gene in embryo kidney samples from the two groups of sheep using the 2¯^ΔΔCt^ method described above.

## Results

### Characterization of the full-length ovine *EDN3* cDNA

**Fig. 2:**
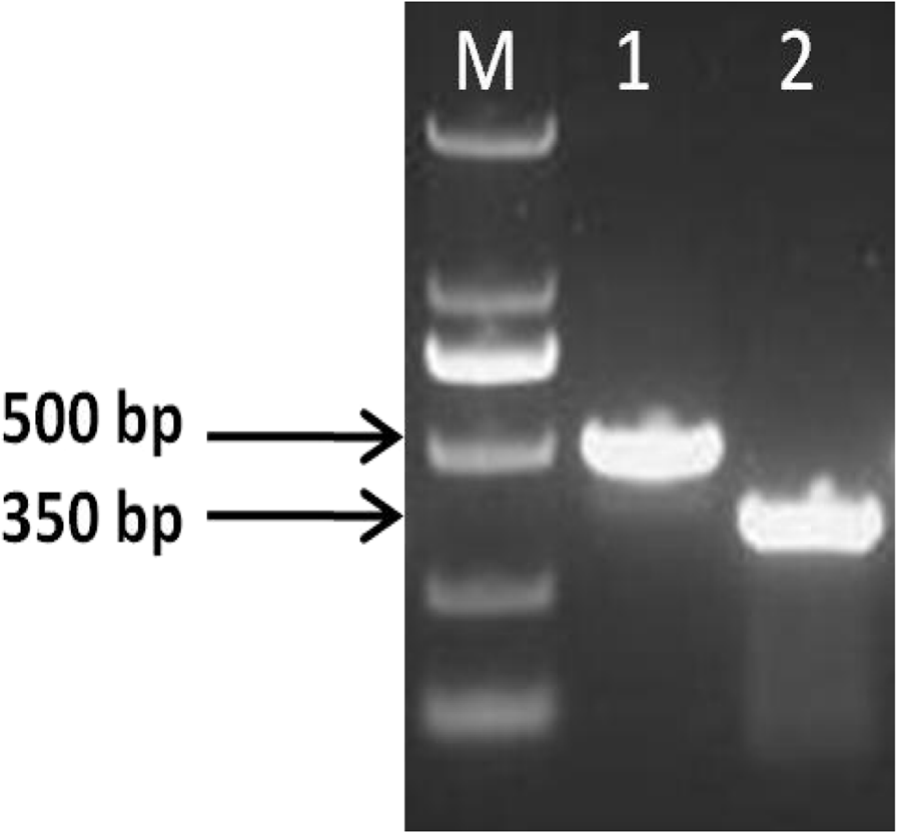
RACE products for sheep *EDN3* gene. 1: for 5′ end; 2: for 3′ end, M: DL2000 DNA Marker

The PCR products from the liver tissue were 1,235 bp. The products obtained from the RACE experiment were 500 bp and 350 bp for the 5′- and 3′-fragments, respectively (Fig. 2).

The data obtained by GenScan software showed that the full length of *EDN3* mRNA was 1,578 bp including 426 bp of 5′ UTR, an open reading frame (ORF) of 639 bp encoding a protein of 212 amino acids, and 513 bp of 3′ UTR. The results obtained using NCBI BLAST tool indicated that the protein sequence deduced from ovine *EDN3* gene has differences in the extent of similarities with that of other species. The similarities observed in the sequences were as follows: goat 99% (Accession No: XP_013824266), cattle 72% (Accession No: NP_001095449.1), pig 70% (Accession No: BAF62297.1), giant panda 70% (Accession No: XP_002915579.1), dog 69% (Accession No: NP_001002942.1), horse 64% (Accession No: XP_001491696.3), Bolivian squirrel monkey 61% (Accession No: XP_003932691.1), chicken 59% (Accession No:BAE45237.1), human 58% (Accession No: AAZ03610), mouse 55% (Accession No:NP_031929), and rat 53% (Accession No: NP_001071118.1). Multiple amino acids sequence alignments of *EDN3* gene were performed and data are shown in Additional file 1: Fig. S1.

As the sheep EDN3 secondary structure is not known, the predicted secondary structure should be used instead. Predicted secondary structure of EDN3 consisted of alpha helix (16.51%), extended strand (20.75%), beta turn (10.85%), and random coil (51.89%; Additional file 1: Fig. S2).

**Fig. 3:**
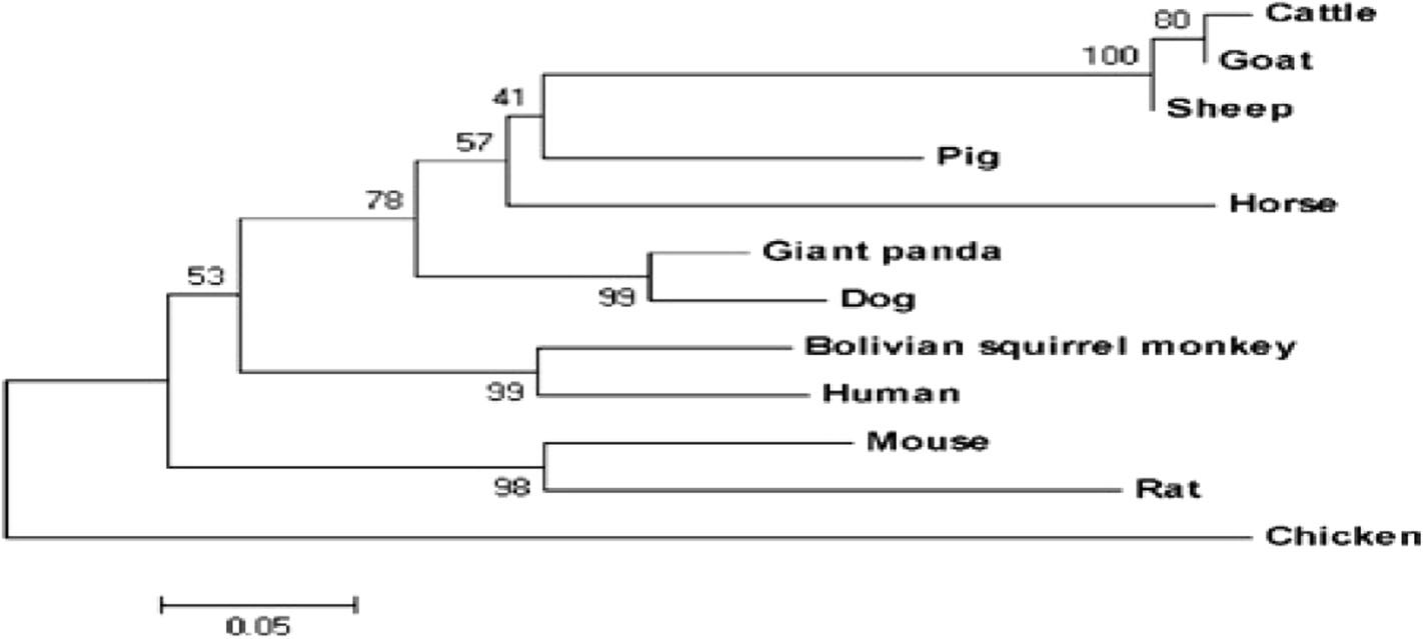
Phylogenetic tree constructed by NJ (Neighbor –Joining) tool. The numbers in the phylogram nodes indicate percent bootstrap support for the phylogeny. The bar at the bottom indicates 5% amino acid divergence in sequence

The putative amino acid sequence of EDN3 had a molecular weight of 29.65 k Dalton (kDa) and the theoretical isoelectric point (pI) of 9.49. Prediction for localization of the protein showed that 47.8% of the protein is nuclear, 21.7% cytoplasmic, 8.7% mitochondrial, 8.7% cytoskeletal, 4.3% in secretory vesicles, 4.3% in the extracellular region, including cell wall, and 4.3% in the Golgi complex. The constructed phylogenetic tree revealed that sheep EDN3 had a close identity with EDN3 of goat and cattle (Fig. 3).

### SNP screening

Four primer pairs 2, 3, 6, and 8 showed 14 polymorphisms on specific genomic regions based on sequencing results of the two DNA pools and the two groups of black bone and non-black bone sheep individuals. All of these SNPs were synonymous and caused no change in the amino acid sequence. The polymorphisms and their location on the chromosome are shown in Additional file 2: Table S1.

### SNP genotyping and association analysis

#### Data processing

From the database available online, we obtained 16 SNPs on chromosome 13, located close to *EDN3*. We assumed that these have a correlation with the trait of our interest. Details of the SNPs are shown in Additional file 2: Table S2. The genomic DNA of 204 individual samples from the two sheep groups was genotyped by using MALDI-TOF. According to the results of the obtained SNPs, we filtered the data to exclude SNPs (*n* = 16) with low signal intensity. Furthermore, 7 and 8 SNPs with genotype call rate < 80% and minor allele frequency < 1% over all individuals were not included in any further analysis in each of the designed crossbreeding and random local populations, respectively.

#### Contributing populations

##### Designed crossbreeding population

The genotype and allele frequency of each SNP were analyzed in black bone (*n* = 22) and non-black bone sheep (*n* = 54) as shown in Table 3. After data filtering, seven SNP sites were identified for genotype distribution analysis. Sites g. 61276174C > T, g. 61276048G > A, g. 61,275,998 T > A, g. 61275916C > T, and g. 61272867G > A were identified by genome screening and sites g. 56451746G > A and g. 56465925C > T by surveying the online database. Genotypes (AA, AG, GG) were detected in both black bone and non-black bone sheep for SNP g. 61272867G > A and g. 56451746G > A, whereas in g. 61276048G > A, the three genotypes were found only in non-black bone sheep, and the genotype GG was absent in black bone sheep. Genotypes (TT, TC, CC) were detected in black bone and non-black bone sheep for SNP g. 61276174C > T. Genotypes (AA, AT, TT) were observed in g. 61,275,998 T > A, whereas genotypes (TT, TC, CC) were recognized in g. 61275916C > T for black bone and non-black bone sheep. Two genotypes (TT, TC) were detected in black bone and non-black bone sheep for SNP g. 56465925C > T. Fisher’s test showed that no significant differences in genotype distributions were observed comparing black bone sheep with non-black bone sheep (*P* > 0.05).

**Table 3:** Genotypes and allele frequency of seven SNPs in the designed crossbreeding population

SNP ID	Breed	Number of samples to each genotype (Genotype frequency, %)			Allele frequency		Fisher’s exact test (*P* value)
		TT	TC	CC	T	C	
g. 61276174C > T	Non-black	23(42.59)	28(51.85)	3(5.56)	0.69	0.31	0.22
	Black	14(63.63)	7(31.82)	1(4.55)	0.80	0.20	
		AA	AG	GG	A	G	
g. 61276048G > A	Non-black	24(44.44)	25(46.30)	5(9.26)	0.68	0.32	0.45
	Black	11(50)	11(50)	0	0.75	0.25	
		AA	AT	TT	A	T	
g. 61,275,998 T > A	Non-black	23(42.59)	28(51.85)	3(5.56)	0.69	0.31	0.16
	Black	14(63.63)	6(31.82)	1(4.55)	0.81	0.19	
		TT	TC	CC	T	C	
g. 61275916C > T	Non-black	23(42.59)	28(51.85)	3(5.56)	0.69	0.31	0.16
	Black	14(63.63)	6(31.82)	1(4.55)	0.81	0.19	
		AA	AG	GG	A	G	
g. 61272867G > A	Non-black	21(38.89)	30(55.55)	3(5.56)	0.67	0.33	0.28
	Black	13(59.09)	8(36.36)	1(4.55)	0.77	0.23	
		AA	AG	GG	A	G	
g. 56451746G > A	Non-black	23(42.59)	28(51.85)	3(5.56)	0.69	0.31	0.22
	Black	14(63.63)	7(31.82)	1(4.55)	0.80	0.20	
		TT	TC	CC	T	C	
g. 56465925C > T	Non-black	47(94.44)	3(5.56)	0	0.97	0.03	0.63
	Black	19(90.90)	2(9.10)	0	0.95	0.05	

##### Random local population

The genotype and allele frequency of each SNP were analyzed in Lanping black bone sheep (*n* = 88) and Lanping non-black bone sheep (*n* = 24) as shown in Table 4. Six SNP sites were identified after cleaning of the genotype data. Three genotypes (AA, AG, GG) were detected in black bone sheep for SNP g. 61276048G > A, g. 61272867G > A, and g. 56451746G > A, whereas only two (AA, AG) were found in non-black bone sheep for the same SNPs. Genotypes (TT, TC, CC) were noticed in black bone sheep for SNP g. 61276174C > T, whereas only two (TT, TC) were found in non-black bone sheep. Genotypes (AA, AT, TT) were detected in black bone sheep for SNP g. 61,275,998 T > A, whereas only two (AA, AT) were found in non-black bone sheep. Genotypes (TT, TC, CC) were detected in black bone sheep for SNP g. 61275916C > T, whereas only two (TT, TC) were found in non-black bone sheep. Fisher’s test showed that no significant differences in genotype distributions were observed comparing black bone sheep with non-black bone sheep (*P* > 0.05).

**Table 4:** Genotypes and allele frequency of six SNPs in the random local population

SNP ID	Breed	Number of samples to each genotype (Genotype frequency, %)			Allele frequency		Fisher’s exact test (*P* value)
		TT	TC	CC	T	C	
g. 61276174C > T	Non-black	19 (87.50)	3(12.50)	0	0.93	0.07	0.19
	Black	80(93.18)	3(3.41)	3(3.41)	0.95	0.05	
		AA	AG	GG	A	G	
g. 61276048G > A	Non-black	19 (87.50)	3(12.5)	0	0.93	0.07	0.19
	Black	80 (93.18)	3(3.41)	3(3.41)	0.95	0.05	
		AA	AT	TT	A	T	
g. 61,275,998 T > A	Non-black	19(87.50)	3(12.5)	0	0.93	0.07	0.24
	Black	82(93.18)	4(4.55)	2(2.27)	0.95	0.05	
		TT	TC	CC	T	C	
g. 61275916C > T	Non-black	19(87.50)	3(12.5)	0	0.93	0.07	0.20
	Black	84(95.45)	3(3.41)	1(1.14)	0.97	0.03	
		AA	AG	GG	A	G	
g. 61272867G > A	Non-black	19(87.50)	3(12.5)	0	0.93	0.07	0.25
	Black	81(92.04)	4(4.55)	3(3.41)	0.94	0.06	
		AA	AG	GG	A	G	
g. 56451746G > A	Non-black	19(87.50)	3(12.5)	0	0.93	0.07	0.20
	Black	84(95.45)	3(3.41)	1(1.14)	0.97	0.03	

### Estimation of variance in *EDN3* copy number in black bone and non-black bone sheep

**Fig. 4:**
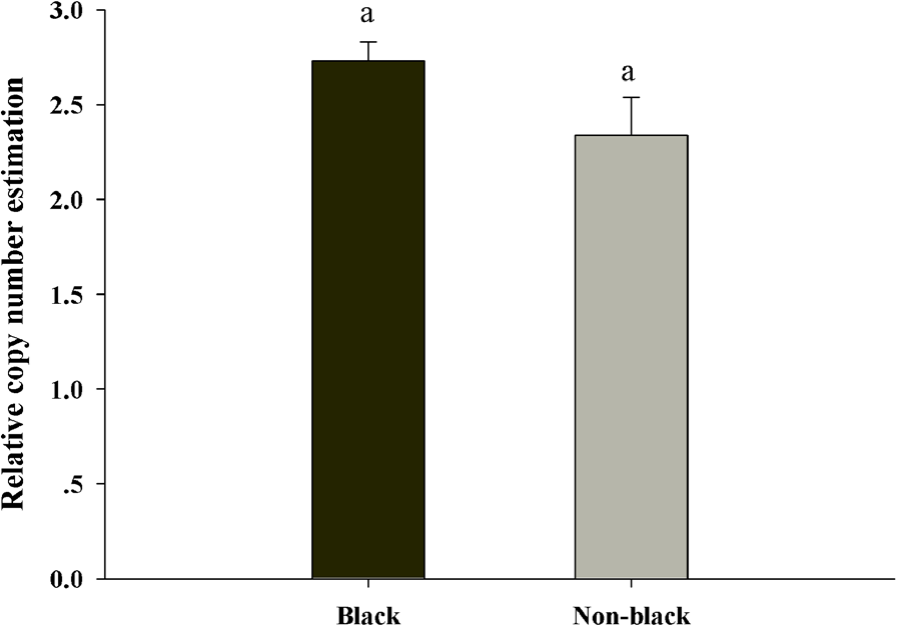
Relative copy number estimation of *EDN3* in black bone and non-black bone sheep. Each bar represents the mean ± S.E. The same letter (a) on the bars denotes insignificant difference within the two groups (*P* > 0.05)

Eight black bone and eight non-black bone sheep were used for detecting the variation in *EDN3* copy number (Fig. 4). High amplification efficiency (95%–105%) with good linearity (*r*^2^ = 0.98) was achieved across the entire concentration range that was created of different dilution series by using genomic DNA. The mean estimate of *EDN3* copy number in the two groups was nearly the same. Welch two sample *t*-test revealed no significant difference between the two groups of sheep (*P* > 0.05).

### Quantitative analysis of *EDN3* mRNA

**Fig. 5:**
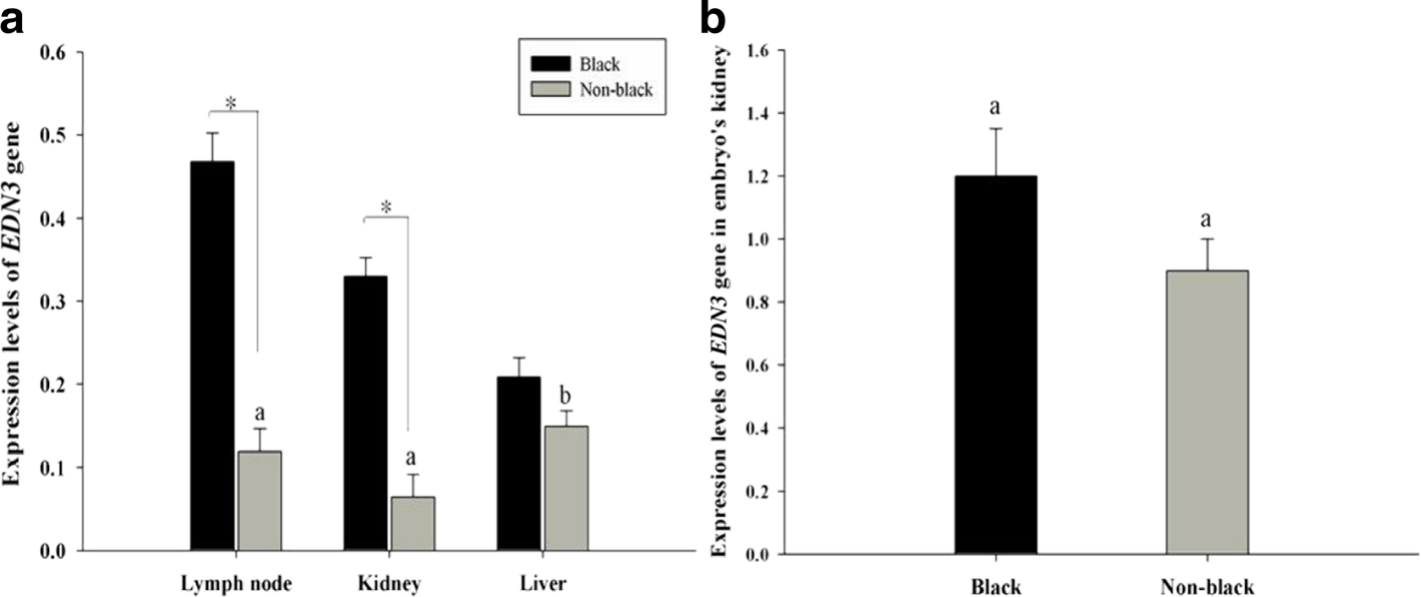
Expression levels of *EDN3* gene among three different tissues in adult black bone and non-black bone sheep (**a**), and in embryo’s kidney (**b**). Note error bars represent the mean ± SE. **a** Letters on bars denote the difference of expression level with significant difference (*P* < 0.05) between liver and lymph node, and extreme significant difference (*P* < 0.01) between liver and kidney. * Significant difference (*P* < 0.05). **b** The same letter (a) on the bars denotes insignificant difference within the two groups (*P* > 0.05)

PCR efficiencies of *EDN3* and *GAPDH* genes were within 95% to 105% that was satisfied for qRT-PCR. The expression levels of *EDN3* mRNA were different among groups and tissues as shown in Fig. 5a.

#### *EDN3* mRNA expression among the groups

Expression levels of *EDN3* gene in lymph node and kidney tissues were significantly higher in black bone sheep than that in non-black bone sheep (*P* < 0.05). Meanwhile, the expression level in liver tissue was insignificantly higher in black bone sheep than that in non-black bone sheep (*P* > 0.05).

#### *EDN3* mRNA expression among tissues

In black bone sheep, the expression levels of *EDN3* varied among the selected tissues and a descending order in expression was observed in lymph node, kidney, and liver. There were no significant differences in the expression levels among the three tissues (*P* > 0.05).

In non-black bone sheep also, the expression levels of *EDN3* varied among the selected tissues and a descending order of expression was observed from liver, lymph node, and kidney. The difference was significant between liver and lymph node (*P* < 0.05), and highly significant between liver and kidney (*P* < 0.01).

### RT-PCR and real-time PCR of *EDN3* in kidney tissue from embryos

**Fig. 6:**
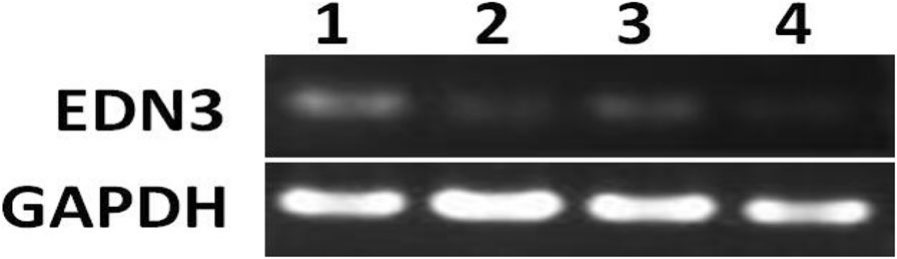
RT-PCR products. 1: kidney from 3 months black bone sheep embryo; 2: from 1 month black bone sheep embryo; 3: from 3 months non-black bone sheep embryo; 4: from 1 month non-black bone sheep embryo

*EDN3* is expressed slightly in sheep embryo’s kidney; the relative expression levels of *EDN3* to *GAPDH* in each kidney sample were 0.1466, 0.0671, 0.1073, and 0.0610 in lane number 1, 2, 3 and 4, respectively (Fig. 6). The difference in the expression of *EDN3* between the two groups of sheep embryo’s kidney is not significant (*P* > 0.05).

The expression level of *EDN3* in embryo’s kidney showed no significant difference between black bone sheep and non-black bone sheep (*P* > 0.05; Fig. 5b).

## Discussion

Researchers have found a suitable method for search and alignment of close species that share high homology and conserved sequences, employing the information from various databases (NCBI, ENSEMBL, and UCSC etc.). RefSeq database (NCBI) provides information about the location of bovine *EDN3* on chromosome 13; the comparative approach by BLAST tool on UCSC genome browser suggested that ovine *EDN3* would map to the same chromosome. Furthermore, *EDN3* mapped to chromosome 2 in mouse [23], chromosome 20 in humans [24], and chromosome 20 in chickens [25]. In the present study, we obtained a partial sequence of *EDN3* from sheep, based on the bovine *EDN3* sequence deposited in NCBI, which displayed a high identity with sheep genome. Thereafter, we performed RACE to obtain the full-length cDNA, the sequence of which was deposited in GenBank (Accession no. KC857456). Interestingly, our data revealed that despite the high identity between EDN3 amino acids in sheep and cattle, the cattle EDN3 showed 60 amino acids that were not observed in sheep EDN3. This demonstrates that every gene has a key function and different evolutionary model, which varies among the species and even the breeds.

We scanned the genomic region (5′-flanking region, all 5 exons, and partial introns) of sheep *EDN3* and found a set of SNPs in black bone and non-black bone sheep. The obtained SNPs and some other SNPs identified by web search, located on chromosome 13, close to *EDN3*, were genotyped to conduct genotype and allele frequency analysis. The results showed that the SNPs found in two different populations of sheep were insignificantly different among the two sheep groups and had no association with the dark trait in black bone sheep. These results might be due to the breed-specific effects and differences in the population size or may be due to incomplete linkage disequilibrium with causal mutations.

To date, little is known about the regulation of *EDN3* CNVs in sheep, especially black bone sheep. Here, we report that genomic copy number analysis in ovine *EDN3* did not show any duplication and was not in accordance with that observed for chicken *EDN3*.

In the current study, we found that *EDN3* was expressed in all of the sampled tissues. Expression levels of *EDN3* mRNA varied between the selected groups as well as among the tissues. Based on the initial studies of Yunnan black bone sheep [2, 26], it has been documented that a large amount of melanin is deposited in black bone sheep compared with non-black bone sheep, and that the melanin content in different tissues of black bone sheep occurs in the order: liver, kidney, spleen, lung, tongue, muscle, skin, and bones, in which was not in a typical conformity with the findings of Muroya et al. [27] in Silky chickens that, the tissues examined were classified significantly in the order of the pigment content as periosteum > gonads (ovary or testis) = trachea ≥ heart, liver, gizzard, cecum, muscles (*Pectoralis* and *Supracoracoideus*), and skin. By visible inspection, there were no differences in coat color for black-bone sheep and non-black bone sheep [2]. As the black pigment distribution in the internal organs of black bone sheep was our trait of interest, in this study, the black internal organs (i.e. lymph node, kidney and liver) were selected to be the targeted research samples for conducting our experiments. *EDN3* gene functions through the entire nervous system (ENS) and multiple roles of *EDN3* were observed during ENS development in the avian hindgut, where it influences neural crest cells (NCCs) proliferation, differentiation, and migration [28]. It was found to be a very potent mitogen and has a role as a survival factor for melanocytes [29]. The findings of Yang et al. [30], revealed that the quantitative mRNA as well as protein expression levels of EDN3 were extremely higher in the skin of black sheep than in white sheep. In a more recent study, Li et al. [31] concluded that *EDN3* promoted melanocytes proliferation from both black and white coat colors, while had no effect on *TYR* gene.

Melanin pigment increases immunity in humans by restricting the growth of human immune deficiency virus in vitro [32] and in vivo [33]. As hyperpigmentation plays an important role in immune system development. Lymph node serves as assistance to the immune system; it works as a filter for harmful substances. Lymph nodes are major sites of B and T lymphocytes and other white blood cells [34]. Herein, significant expression difference of *EDN3* was observed in lymph node tissue between black bone and non-black bone sheep which indicate the link between the lymphocytes and the biological function of EDN3 in neural crest cells migration and proliferation through the entire body, that would be in a great harmony with a previous study showed a highly significant amount of immunoglobulin G (IgG) and immunoglobulin M (IgM) were found in the plasma of black bone and not in non-black bone sheep [2]. These findings might explain the immune role of these potentially valuable sheep.

The expression levels were insignificantly higher in liver of black bone than that in non-black bone sheep. However, a significant expression difference of *EDN3* was observed in kidney between the two groups, which illustrate the regulatory role that *EDN3* would play in the excretory system.

Former researches have reported that *EDN3* plays a role in the early embryonic development [35]. In this experiment, we firstly used the tissues that were obtained from mature sheep. In order to better understand the relative expression patterns, we used embryo kidney sample. However, the result was not concordant with that obtained from mature sheep, which warrants further investigation to detect the origin of the black pigment in a different developmental stages of black bone sheep.

## Conclusions

In summary, we performed a complete cloning of sheep *EDN3* mRNA. Thirty SNPs were detected inside or around the *EDN3* gene, but none of them was significantly associated with dark traits in black bone sheep. Genomic copy number analysis revealed no significant difference between black bone sheep and non-black bone sheep. Differences in the expression pattern of *EDN3* gene were shown among various tissues and groups in sheep, but no specific regulation of expression was observed in liver between black bone sheep and non-black bone sheep. However, the expression difference in the lymph node and kidney is pending further studies to identify the immune and excretory functions that *EDN3* gene regulate in black bone sheep. Therefore, we speculate that *EDN3* gene might not be a key gene for the black trait in sheep as is reported in Silky chicken, but on the other hand, it would be involved in the regulation of immune and urinary systems in black bone sheep.

## Additional files

Additional file 1: **Figure S1.** Alignments of EDN3 protein with different species. **Figure S2.** Prediction secondary structure of EDN3 protein. t, refers to Beta turn; c, to Random coil; h, to Alpha helices; e, to Extended strand. (DOCX 13238 kb)

Additional file 2: **Table S1.** *EDN3* genome sequencing screening results. **Table S2.** The 16 SNPs obtained from online database. OAR refers to: *Ovis aries*; Chr: chromosome. (DOCX 18 kb)

## Abbreviations

CDS: Coding domain sequence
CNVs: Copy number variations
*DGAT1*: Diacylglycerol O-acyltransferase 1
EDN3: Endothelin 3
EDNRB: Endothelin receptor type B
ENS: Entire nervous system
FM: Fibromelanosis
GAPDH: Glyceraldehyde-3-phosphatedehydrogenase
IgG: Immunoglobulin G
IgM: Immunoglobulin M
Kit 1: Kit ligand 1
Kit: KIT proto-oncogene receptor tyrosine kinase
MALDI-TOF MS: Matrix assisted laser desorption-ionization time-of-flight mass spectrometry
MC1R: Melanocortin 1 receptor
MITF: Microphthalmia-associated transcription factor
NCCs: Neural crest cells
ORF: Open reading frame
qRT-PCR: Quantitative real-time PCR
RACE: Rapid amplification of cDNA ends
RT-PCR: Reverse transcription PCR
SNPs: Single nucleotide polymorphisms
TRP1: Tyrosinase-related protein 1
TRP2: Tyrosinase-related protein 2
TYR: Tyrosinase
UTR: Untranslated region
