# Supplementary material for: Molecular cloning and characterization of the endothelin 3 gene in black bone sheep
Source: J Anim Sci Biotechnol. 2018 Jun 25;9:57. doi: 10.1186/s40104-018-0272-y (PMC6022492; doi:10.1186/s40104-018-0272-y)

**
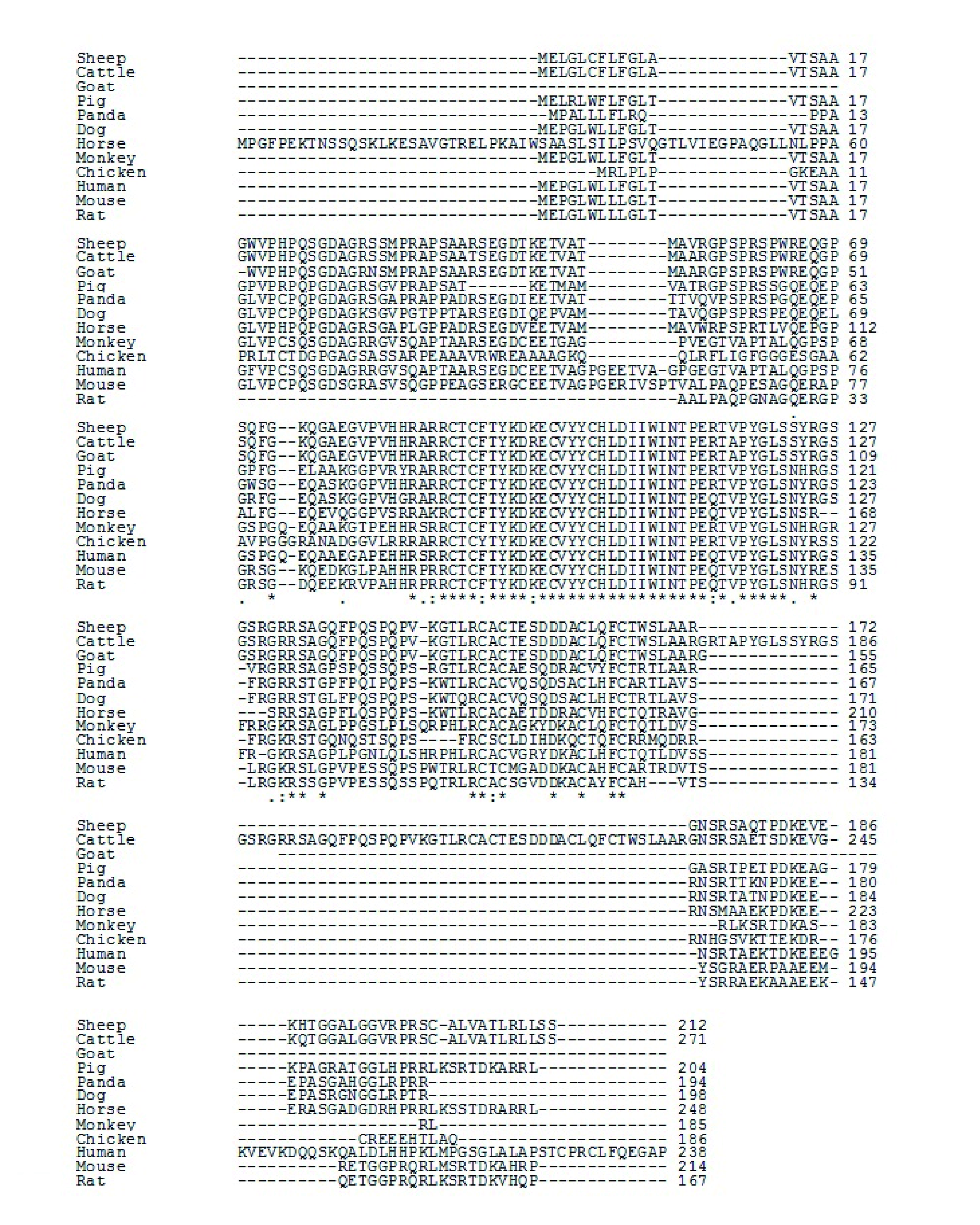
Additional file 1: Figure S1.** Alignments of *EDN3* protein with different species

**Figure S2.** Prediction secondary structure of *EDN3* Protein

t, refers to Beta turn; c, to Random coil; h, to Alpha helices; e, to Extended strand


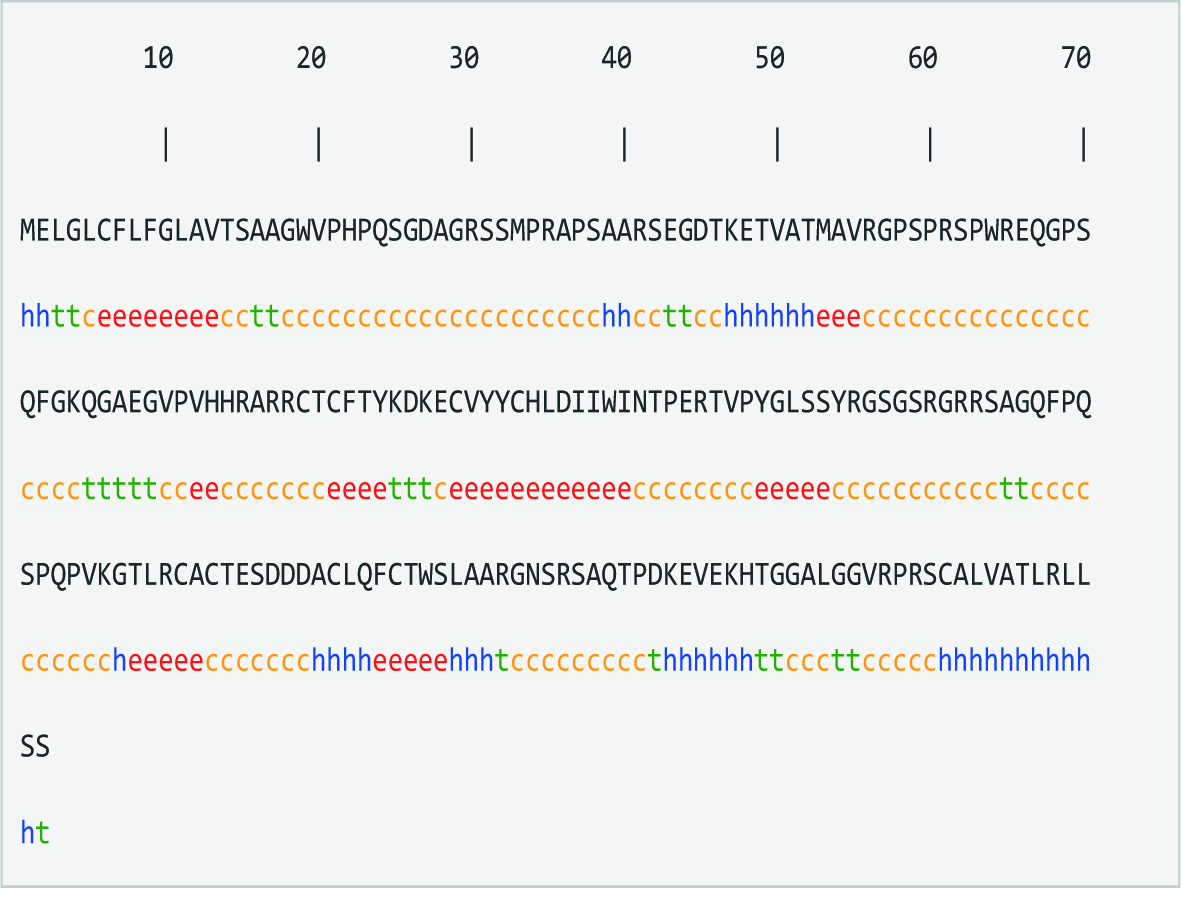

Supplement: Supplementary file 1 — Figure S1. Alignments of EDN3 protein with different species. Figure S2. Prediction secondary structure of EDN3 protein. t, refers to Beta turn; c, to Random coil; h, to Alpha helices; e, to Extended strand. (DOCX 13238 kb) [file 40104_2018_272_MOESM1_ESM.docx]
