# Supplementary material for: Molecular cloning and characterization of the endothelin 3 gene in black bone sheep
Source: J Anim Sci Biotechnol. 2018 Jun 25;9:57. doi: 10.1186/s40104-018-0272-y (PMC6022492; doi:10.1186/s40104-018-0272-y)
Supplement: Supplementary file 2 — Table S1. EDN3 genome sequencing screening results. Table S2. The 16 SNPs obtained from online database. OAR refers to: Ovis aries; Chr: chromosome. (DOCX 18 kb) [file 40104_2018_272_MOESM2_ESM.docx]

**Additional file 2: Table S1.** The 14 SNPs obtained from *EDN3* genome screening

| **Primer No.** | **SNP ID** | **Black bone sheep** | **Non-black bone sheep** | **Pool DNA** | | **Reference**  **(UCSC)** | **Location** |
| --- | --- | --- | --- | --- | --- | --- | --- |
|  |  |  |  | **Black bone sheep** | **Non-black bone sheep** |  |  |
| 1 | -- | -- | -- | -- | -- | -- | No polymorphism |
| 2 | g. 61296262T>C  g. 61296200T>C  g. 61296048A>G | T  C  A | C  T  G | T  T  A | T  T  A | T  T  N | 61296262 Exon 1  61296200 Exon 1  61296048 Intron 1 |
| 3 | g. 61295848T>C  g. 61295757A>G  g. 61295684T>C  g. 61295591A>G  g. 61295576C>T | C  A  C  G  T | T  G  T  A  C | T  A  T  A  C | T  A  T  A  C | T  A  T  A  C | 61295848 Intron 1  61295757 Intron 1  61295684 Intron 1  61295591 Exon 2  61295576 Exon 2 |
| 4 | -- | -- | -- | -- | -- | -- | No polymorphism |
| 5 | -- | -- | -- | -- | -- | -- | No polymorphism |
| 6 | g. 61276174C>T  g. 61276048G>A  g. 61275998T>A  g. 61275990C>T  g. 61275916C>T | T  A  A  T  T | C>T  G>A  T>A  C>T  C>T | T  A  A  T  T | C >T  G>A  T>A  C>T  C>T | C  G  T  C  C | 61276174 Exon 3  61276048 Intron 3  61275998 Intron 3  61275990 Intron 3  61275916 Intron 3 |
| 7 | -- | -- | -- | -- | -- | -- | No polymorphism |
| 8 | g. 61272867G>A | A | G>A | A | G>A | G | 61272867 Intron 4 |

**Table S2.** The 16 SNPs obtained from online database

OAR refers to: *Ovis aries*; Chr: chromosome

| SNP ID | SNP  genotype | Chr | Location |
| --- | --- | --- | --- |
| g. 56421467A>C | C/A | OAR13 | 56421467 |
| g. 56448788G>A | A/G | OAR13 | 56448788 |
| g. 56450128T>C | C/T | OAR13 | 56450128 |
| g. 56451746G>A | A/G | OAR13 | 56451746 |
| g. 56453781A>G | G/A | OAR13 | 56453781 |
| g. 56454854T>G | G/T | OAR13 | 56454854 |
| g. 56456767G>A | A/G | OAR13 | 56456767 |
| g. 56458523T>C | C/T | OAR13 | 56458523 |
| g. 56459679A>G | G/A | OAR13 | 56459679 |
| g. 56460313T>C | C/T | OAR13 | 56460313 |
| g. 56461719C>A | A/C | OAR13 | 56461719 |
| g. 56463927G>T | T/G | OAR13 | 56463927 |
| g. 56464833G>T | T/G | OAR13 | 56464833 |
| g. 56465925C>T | T/C | OAR13 | 56465925 |
| g. 56466846A>C | C/A | OAR13 | 56466846 |
| g. 56550527A>C | C/A | OAR13 | 56550527 |
